# Receipt of Targeted Therapy and Survival Outcomes in Patients With Metastatic Colorectal Cancer

**DOI:** 10.1001/jamanetworkopen.2022.50030

**Published:** 2023-01-19

**Authors:** Siran M. Koroukian, Benjamin D. Booker, Long Vu, Fredrick R. Schumacher, Johnie Rose, Gregory S. Cooper, J. Eva Selfridge, Sarah C. Markt

**Affiliations:** 1 Department of Population and Quantitative Health Sciences, Case Western Reserve University School of Medicine, Cleveland, Ohio; 2 Case Comprehensive Cancer Center, Case Western Reserve University School of Medicine, Cleveland, Ohio; 3 Center for Community Health Integration, School of Medicine, Case Western Reserve University, Cleveland, Ohio; 4 Department of Internal Medicine, University Hospitals Cleveland Medical Center, Cleveland, Ohio; 5 Division of Solid Tumor Oncology, University Hospitals Cleveland Medical Center, Cleveland, Ohio

## Abstract

**Importance:**

Professional society guidelines recommend treating patients with metastatic colorectal cancer with targeted therapies, including epithelial growth factor receptor (EGFR) inhibitors and vascular endothelial growth factor (VEGF) inhibitors, depending on the presence or absence of certain mutations. Since most studies of first-line targeted therapies have been limited by sample size, there is a need for larger studies using data from routine clinical care.

**Objectives:**

To identify factors associated with receipt of first-line targeted therapies among patients with metastatic colorectal cancer for whom *RAS* or *BRAF* mutation data in the tumor were available and investigate whether targeted therapy is associated with survival.

**Design, Setting, and Participants:**

This cohort study used deidentified data from an electronic health record–derived database to include patients from 800 sites of patient care across the US who were diagnosed with de novo metastatic colorectal cancer between January 1, 2013, and March 31, 2020 (n = 9134).

**Main Outcomes and Measures:**

Receipt of first-line targeted therapy, categorized as ever having received EGFR inhibitors, VEGF inhibitors, or neither. The secondary outcome was overall survival.

**Results:**

The study population included 9134 patients. The median age at diagnosis was 62 years (IQR, 53-71 years), 5019 (54.9%) were male, and 5692 (62.3%) were White. The median follow-up period was 15 months. Overall, 713 patients (7.8%) received EGFR inhibitors and 5081 patients (55.6%) received VEGF inhibitors as part of their first-line treatment. Among patients with *RAS* wild-type (*RAS*-WT) tumors, 625 patients (15.5%) received EGFR inhibitors and 2053 patients (50.9%) received VEGF inhibitors. In patients with *RAS* mutant (*RAS-*Mut) tumors, 50 patients (1.1%) received EGFR inhibitors and 2682 patients (59.7%) received VEGF inhibitors; among those with *BRAF*-mutant (*BRAF*-Mut) tumors, 38 patients (6.3%) received EGFR inhibitors and 346 patients (57.2%) received VEGF inhibitors. More than one-third of the patients (36.6%) received neither EGFR inhibitors nor VEGF inhibitors. Compared with patients younger than age 40 years, those aged 80 years or older had significantly lower odds to receive targeted therapies (EGFR or VEGF inhibitors in patients with *RAS-*WT tumors: adjusted odds ratio [aOR], 0.53; 95% CI, 0.36-0.79; and VEGF inhibitors in patients with *RAS*-Mut tumors: aOR, 0.62; 95% CI, 0.42-0.90). Improved survival was associated with EGFR inhibitor therapy in patients with *RAS-*WT tumors (adjusted hazard ratio [aHR], 0.85; 95% CI, 0.74-0.98). Unlike in clinical trials, however, no survival benefit was noted with use of VEGF inhibitors among patients with *RAS*-WT (aHR, 1.00; 95% CI, 0.91-1.11) or *RAS*-Mut (aHR, 1.01; 95% CI, 0.93-1.10) tumors.

**Conclusions and Relevance:**

The findings of this study showed mixed results on survival benefits associated with targeted therapy. In addition, given that some of the results differed from those of randomized clinical trials, this study highlights the importance of using data originating from routine clinical care.

## Key Points

**Question:**

What are the factors and survival outcomes associated with receipt of guideline-recommended targeted therapies in patients with metastatic colorectal cancer?

**Findings:**

In this cohort study of 9134 patients, more than one-third of the patients received no targeted therapy. The findings showed survival benefits associated with receipt of epithelial growth factor receptor inhibitors, but only in patients with *RAS–*wild type tumors; certain combinations of targeted therapy and chemotherapy were associated with improved survival in smaller patient subgroups.

**Meaning:**

The findings of this study showed mixed results on survival benefits associated with targeted therapy; given the differences between these findings and those of randomized clinical trials, this study highlights the importance of analyzing outcomes in routine clinical practice.

## Teaser

This cohort study examines the use of targeted therapies, including epithelial growth factor receptor inhibitors and vascular endothelial growth factor inhibitors, in the treatment of patients with metastatic colorectal cancer.

## Introduction

Colorectal cancer (CRC) is the third most common cancer in US men and women, claiming over 52 000 lives in 2021 alone.^1^ While CRC is amenable to screening, 21% of patients with CRC are diagnosed with metastatic disease, with poor prognosis. The 5-year relative survival is 90% among patients diagnosed with localized CRC, but 14% among those diagnosed with metastatic CRC (mCRC).^2^

Molecular profiling of *RAS* (*KRAS*, *NRAS*) and *BRAF* mutations has revolutionized the treatment of mCRC, as evidenced through guidelines by the National Comprehensive Cancer Network and several other professional societies.^3,4,5,6^ Not surprisingly, therefore, recent studies have reported increasing trends in the use of molecular testing in patients with mCRC.^7,8^

The presence or absence of certain variations informs the choice of targeted therapies by such agents as epithelial growth factor receptor (EGFR) inhibitors, which are effective in *RAS* wild-type (WT) left-sided mCRC tumors, and vascular endothelial growth factor (VEGF) inhibitors, used regardless of molecular subtypes.^9^ A pooled analysis of 33 randomized clinical trials (RCTs) including 15 025 patients with mCRC concluded that EGFR inhibitor therapy was associated with improved progression-free and overall survival.^10^ Another pooled analysis from 4 RCTs and 1539 patients with mCRC similarly showed survival benefits among those with left-sided, but not right-sided, tumors.^11^ Furthermore, among patients with *RAS-*WT tumors, studies have shown survival advantage with the addition of EGFR inhibitors to a chemotherapy backbone, such as FOLFOX (folinic acid, fluorouracil, and oxaliplatin)^12^ or FOLFIRI (folinic acid, fluorouracil, and irinotecan).^13^ However, other studies have shown no survival benefits associated with EGFR inhibitor therapy in patients with a *KRAS*-WT tumor^14^ or varying efficacy of EGFR inhibitors by chemotherapy backbone.^15^

With VEGF inhibitors, a meta-analysis of 7 RCTs that included 2040 patients with mCRC reported that VEGF inhibitor therapy was associated with improved progression-free survival time, but not with overall survival.^16^ Another study pooling data from 9 trials comprising 3914 patients noted that the addition of VEGF inhibitors to various chemotherapy regimens was associated with higher progression-free survival, as well as higher overall survival.^17^

Data gathered from RCTs reflect efficacy from regimented treatment administered to patients who tend to be relatively young and healthy. Large population-based studies are needed to determine whether findings from RCTs can be translated to benefits in routine clinical care.^18^ Previous observational studies are based on small numbers of patients, with mixed results.^19,20^ One of the larger studies on bevacizumab, a VEGF inhibitor, including 2526 patients with mCRC and using data from the 2002-2007 Surveillance, Epidemiology, and End Results–Medicare linked database, reported small improvements in overall survival and did not include information on mutation status.^21^

The purpose of this study was 2-fold: first, to identify factors, primarily mutational status, associated with receipt of first-line targeted therapies among patients with mCRC, and second, to describe the association of first-line targeted therapy with survival. We address the limitations from previous studies by using 2013-2020 data originating from electronic health records including more than 9000 patients with mCRC receiving care in community and academic practice sites in routine clinical care.

## Methods

### Study Population

We used the nationwide Flatiron Health deidentified electronic health record–derived database, comprising patient-level structured and unstructured data curated via technology-enabled abstraction, originating from more than 800 sites of patient care across the US.^22,23^ We selected 14 480 patients diagnosed with de novo mCRC between January 1, 2013, and March 31, 2020, with at least 6 months of follow-up after diagnosis. The maximum follow-up period was 90 months. We excluded 436 patients with unknown sex (≤5) or unknown tumor site (colon or rectum), 2243 patients who did not have documented treatment information, those who tested positive for more than 1 biomarker (*KRAS*, *NRAS*, *BRAF*; n = 118) and had *RAS* and *BRAF* mutation status unknown (n = 2481), and those who received both EGFR inhibitors and VEGF inhibitors as first-line treatment (n = 68). Our final study population included 9134 patients (eFigure 1 in Supplement 1). Our study followed the Strengthening the Reporting of Observational Studies in Epidemiology (STROBE) reporting guideline. This study was deemed to not include human participants by the Case Western Reserve University institutional review board. Accordingly, and per federal regulations (45 CFR §46.102), it was not required that we obtain consent for research.

### Treatment Ascertainment

We defined first-line targeted therapies (EGFR or VEGF inhibitors) based on documentation of receipt in the medical record. Patients who had received panitumumab or cetuximab were classified as having received EGFR inhibitor therapy. Patients who had received bevacizumab, ramucirumab, and ziv-aflibercept were classified as having received VEGF inhibitor therapy. Patients who did not receive EGFR inhibitors or VEGF inhibitors were categorized as having received no targeted therapy.

We also identified first-line treatment regimens as defined by business rules that were received by at least 80 patients in a given chemotherapy backbone. The threshold of 80 patients was based on the distribution of patients across the treatment regimens. Regimens that were received by fewer than 80 patients were classified into other categories according to the chemotherapy backbone (FOLFOX + other, FOLFIRI + other, FOLFOXIRI + other, capecitabine + other, and other regimen).

### Outcomes of Interest

For our first aim, to identify factors associated with receipt of targeted therapy, we categorized receipt of targeted therapy as receipt of an EGFR inhibitor or a VEGF inhibitor, compared with neither. For our second aim, overall survival was our primary outcome of interest, defined as the time from receipt of first-line treatment to date of death from any cause (censored to the first of the month of death). Patients without a date of death were censored at their last visit.

### Tumor Characteristics and Mutation Status

Patients’ tumors were classified in 3 mutually exclusive categories (eTable 1 in Supplement 1): *RAS* mutant (*RAS*-Mut) if they ever had documentation of a positive test for a *KRAS* or *NRAS* mutation (n = 4495), with *BRAF* mutation status being negative or unknown; *BRAF* mutant (*BRAF*-Mut) if they ever had documentation of a positive test for a *BRAF* mutation (n = 605) with *RAS* mutation status being negative or unknown; and *RAS-*WT if they had documentation of negative tests for *KRAS* and had *NRAS* and *BRAF* negative or unknown status (n = 4034). Because *RAS* and *BRAF* mutation are generally thought to be mutually exclusive, we assumed that mutations of unknown status were in fact negative.

Other tumor characteristics included the site of the primary tumor (rectum or colon), tumor group stage, and mismatch repair deficiency status (microsatellite instable, microsatellite stable, or unknown). Patients were classified as being microsatellite instable if their medical records ever documented microsatellite instable–high or loss of mismatch repair deficiency protein expression.

### Covariates

Additional independent variables included patient demographic characteristics, payer type, clinical setting, comorbidity burden, and Eastern Cooperative Oncology Group (ECOG) score. Baseline demographic and clinical variables included age at metastatic diagnosis (<40, 40-49, 50-59, 60-69, 70-79, ≥80 years), sex (male, female), and race and ethnicity (African American or Black [hereafter, Black], Asian, Hispanic or Latinx [hereafter, Hispanic], White, and other [ie, with a race category not listed in the aforementioned categories]), as documented in the electronic health records that can be self-reported by the patient^22^ or categorized by clinical staff. We identified patients as Hispanic or Latinx if ethnicity or race was reported as Hispanic regardless of the race category. Payer category (commercial, Medicare, other government program, and unknown) was defined as the payer with the closest start date before metastatic diagnosis that had an end date postdiagnosis or no end date listed in the database. Distinct comorbidities, as defined by Charlson et al,^24^ were assessed using *International Classification of Diseases, 9th Edition*, and *International Statistical Classification of Diseases, 10th Edition*, diagnostic codes recorded 6 months before or after metastatic diagnosis and dichotomized based on their Charlson index score (≤mean +1 SD or >mean +1 SD). Functional status was defined as the median ECOG performance score, from scores recorded 6 months before or after metastatic diagnosis, rounded up. ECOG level was categorized as 0 or 1 (little to no impairment), 2 (some impairment), 3 or 4 (high impairment), and missing. We accounted for these covariates, including race and ethnicity, to analyze patterns of treatment and survival outcomes by patient characteristics.

### Statistical Analysis

Demographic, clinical, and tumor characteristics were compared by receipt of EGFR inhibitors, VEGF inhibitors, or no targeted therapy. We also compared receipt of first-line therapy, including all regimens, by mutation status. To compare factors associated with receipt of targeted therapy, we conducted multivariable logistic regression models, stratified by mutation status. In patients with *RAS*-WT tumors, receipt of EGFR inhibitors or VEGF inhibitors is indicated by National Comprehensive Cancer Network guidelines^3^; therefore, we evaluated clinical and demographic factors associated with receipt of either EGFR inhibitors or VEGF inhibitors, compared with neither. We conducted similar analyses among patients with *RAS*-Mut tumors to evaluate the odds of receiving VEGF inhibitors, compared with those who did not receive VEGF inhibitors (including EGFR inhibitors or nontargeted therapies).

We evaluated the association between targeted therapies and overall survival, comparing receipt of EGFR and VEGF inhibitors with receipt of neither, using Cox proportional hazards models and adjusted survival curves.^25^ We conducted these analyses separately (1) among patients with *RAS*-WT tumors, (2) among patients with *RAS-*Mut tumors, and (3) among patients with *BRAF*-Mut tumors. We conducted similar analyses to estimate the association between specific treatment regimens and overall survival.

For all models, missing values were modeled using the missing indicator approach in order to not exclude these observations. We used R, version 4.0.5 (R Foundation for Statistical Computing) for all statistical analysis, with a 2-tailed α level of .05 as the significance threshold.

## Results

Our study population included 9134 patients. The median follow-up period was 15 months, the median age was 62 years (IQR, 53-71 years), 5019 (54.9%) were male, and 4115 (45.1%) were female (Table 1). Three percent of the patients were Asian, 10.8% were Black, 7.5% were Hispanic, 62.3% were White, and 16.4% were of other or unknown race. The percentage of Black patients was higher among those receiving VEGF inhibitors (12.0%) than among those receiving EGFR inhibitors (9.0%) or neither (9.3%). The percentage of women was considerably lower among those receiving EGFR inhibitors (38.3%) than among those receiving VEGF inhibitors (45.3%) or no targeted therapies (46.1%). Over half of the patients (56.3%) had an ECOG score of 0 or 1, and 35.3% had a missing ECOG score; however, the percentage of patients with an ECOG score of 0 or 1 was higher among those receiving VEGF inhibitors (60.8%) than among those receiving EGFR inhibitors (51.6%) or no targeted therapies (50.5%). A higher percentage of patients with rectal cancer was in the EGFR inhibitor (22.7%) or no targeted therapies (24.6%) groups than in the VEGF inhibitor group (17.6%).

**Table 1.** Demographic and Clinical Characteristics of Patients With mCRC by Receipt of EGFR Inhibitors, VEGF Inhibitors, or Neither as First-Line Therapy, Flatiron Health Database 2013-2020

Characteristic	No. (%)^a^			
	Overall (n = 9134)	EGFR inhibitor (n = 713)	VEGF inhibitor (n = 5081)	Neither EGFR nor VEGF inhibitor (n = 3340)
Age, y				
<40	454 (5.0)	31 (4.3)	251 (4.9)	172 (5.1)
40-49	1187 (13.0)	110 (15.4)	666 (13.1)	411 (12.3)
50-59	2277 (24.9)	187 (26.2)	1336 (26.3)	754 (22.6)
60-69	2611 (28.6)	211 (29.6)	1491 (29.3)	909 (27.2)
70-79	2043 (22.4)	142 (19.9)	1104 (21.7)	797 (23.9)
≥80	562 (6.2)	32 (4.5)	233 (4.6)	297 (8.9)
Age at diagnosis, median (IQR), y	62 (53-71)	61 (51-69)	62 (52-70)	63 (53-73)
Race and ethnicity				
African American or Black	985 (10.8)	64 (9.0)	609 (12.0)	312 (9.3)
Asian	272 (3.0)	21 (2.9)	165 (3.2)	86 (2.6)
Hispanic or Latinx	686 (7.5)	60 (8.4)	375 (7.4)	251 (7.5)
White	5692 (62.3)	439 (61.6)	3143 (61.9)	2110 (63.2)
Other^b^	719 (7.9)	58 (8.1)	384 (7.6)	277 (8.3)
Missing	780 (8.5)	71 (10.0)	405 (8.0)	304 (9.1)
Sex				
Female	4115 (45.1)	273 (38.3)	2303 (45.3)	1539 (46.1)
Male	5019 (54.9)	440 (61.7)	2778 (54.7)	1801 (53.9)
ECOG score				
0 or 1	5147 (56.3)	368 (51.6)	3091 (60.8)	1688 (50.5)
2	646 (7.1)	<50 (<10)	>310 (>6.0)	283 (8.5)
3 or 4	120 (1.3)	≤5	>50 (≤5)	62 (1.9)
Missing	3221 (35.3)	294 (41.2)	1620 (31.9)	1307 (39.1)
Charlson Comorbidity Index score				
≤Mean +1 SD	7969 (87.2)	638 (89.5)	4405 (86.7)	2926 (87.6)
>Mean +1 SD	1165 (12.8)	75 (10.5)	676 (13.3)	414 (12.4)
Stage				
IV	2253 (24.7)	174 (24.4)	1215 (23.9)	864 (25.9)
IVA	4164 (45.6)	345 (48.4)	2300 (45.3)	1519 (45.5)
IVB	2430 (26.6)	168 (23.6)	1409 (27.7)	853 (25.5)
IVC	287 (3.1)	26 (3.6)	157 (3.1)	104 (3.1)
Tumor site				
Colon	7253 (79.4)	551 (77.3)	4185 (82.4)	2517 (75.4)
Rectum	1881 (20.6)	162 (22.7)	896 (17.6)	823 (24.6)
MMR status				
MSS	6307 (69.0)	497 (69.7)	3571 (70.3)	2239 (67.0)
MSI	325 (3.6)	22 (3.1)	166 (3.3)	137 (4.1)
Unknown/missing	2502 (27.4)	194 (27.2)	1344 (26.5)	964 (28.9)
Variation				
*BRAF*-Mut	605 (6.6)	38 (5.3)	346 (6.8)	221 (6.6)
*RAS*-Mut	4495 (49.2)	50 (7.0)	2682 (52.8)	1763 (52.8)
*RAS*-WT	4034 (44.2)	625 (87.7)	2053 (40.4)	1356 (40.6)

Abbreviations: ECOG, Eastern Cooperative Oncology Group; EGFR, epithelial growth factor receptor; mCRC, metastatic colorectal cancer; MMR, mismatch repair deficiency; MSI, microsatellite instable; MSS, microsatellite stable; Mut, mutation; VEGF, vascular endothelial growth factor; WT, wild-type.

^a^ In accordance with our data use agreement, cell sizes less than or equal to 5 were masked. Additional cells in corresponding rows and columns were also approximated to prevent derivation of the masked numbers.

^b^ Other includes patients whose race and ethnicity is not listed in the specified categories.

In our study population, 6.6% of the patients had a *BRAF*-Mut, 49.2% had *RAS*-Mut, and 44.2% were *RAS*-WT. Since patients with *RAS*-Mut tumors do not benefit from EGFR inhibitor therapy, the percentage of patients with *RAS*-Mut tumors was much lower in patients receiving EGFR inhibitors than among those receiving VEGF inhibitors or among those who received no targeted therapies (7.0%, compared with 52.8% in each of the other 2 groups). Conversely, a much higher percentage of patients receiving EGFR inhibitor therapy had *RAS*-WT tumors (87.7%), compared with 40.4% among patients receiving VEGF inhibitor therapy and 40.6% among those with no targeted therapies.

Overall, 713 patients (7.8%) received EGFR inhibitors and 5081 patients (55.6%) received VEGF inhibitors as part of their first-line treatment, regardless of chemotherapy backbone (Table 2). Among patients with *RAS*-WT tumors, 15.5% received EGFR inhibitors and 50.9% received VEGF inhibitors, regardless of chemotherapy backbone. Among patients with an *RAS*-Mut tumor, 1.1% received EGFR inhibitors and 59.7% received VEGF inhibitors and, among those with a *BRAF*-Mut tumor, 6.3% received EGFR inhibitors and 57.2% received VEGF inhibitors. More than one-third (36.6%) of the patients received neither EGFR inhibitor nor VEGF inhibitor therapy (Table 2). The most common first-line treatment regimens were FOLFOX + VEGF inhibitor (36.6%), FOLFOX alone (15.9%), capecitabine alone (7.2%), and FOLFIRI + VEGF inhibitor (5.0%) (Table 2). This ranking was similar across the groups stratified by mutation status.

**Table 2.** Receipt of First-Line Treatment by Mutation Status

Characteristic	Patients, No. (%)^a^			
	Overall (n = 9134)	*RAS*-WT (n = 4034)	*RAS*-Mut (n = 4495)	*BRAF*-Mut (n = 605)
Any EGFR inhibitor				
Yes	713 (7.8)	625 (15.5)	50 (1.1)	38 (6.3)
No	8421 (92.2)	3409 (84.5)	4445 (98.9)	567 (93.7)
Any VEGF inhibitor				
Yes	5081 (55.6)	2053 (50.9)	2682 (59.7)	346 (57.2)
No	4053 (44.4)	1981 (49.1)	1813 (40.3)	259 (42.8)
Neither EGFR inhibitor nor VEGF inhibitor	3340 (36.6)	1356 (33.6)	1763 (39.2)	221 (36.5)
Other regimens				
FOLFOX, VEGF inhibitor	3340 (36.6)	1366 (33.9)	1746 (38.8)	228 (37.7)
Capecitabine	661 (7.2)	273 (6.8)	351 (7.8)	37 (6.1)
With VEGF inhibitor	151 (1.7)	53 (1.3)	82 (1.8)	16 (2.6)
With other	71 (0.8)	38 (0.9)	>30 (≤5)	≤5
CAPEOX	196 (2.1)	89 (2.2)	96 (2.1)	11 (1.8)
With VEGF inhibitor	360 (3.9)	159 (3.9)	179 (4.0)	22 (3.6)
Clinical study drug	156 (1.7)	54 (1.3)	87 (1.9)	15 (2.5)
Fluorouracil	114 (1.2)	62 (1.5)	>50 (≤5)	≤5
With Leucovorin	91 (1.0)	36 (0.9)	49 (1.1)	6 (1.0)
With Leucovorin and VEGF inhibitor	142 (1.6)	50 (1.2)	86 (1.9)	6 (1.0)
FOLFIRI	202 (2.2)	67 (1.7)	117 (2.6)	18 (3.0)
With EGFR inhibitor	180 (2.0)	155 (3.8)	17 (0.4)	8 (1.3)
With VEGF inhibitor	456 (5.0)	161 (4.0)	265 (5.9)	30 (5.0)
With other	48 (0.5)	21 (0.5)	>20 (≤5)	≤5
FOLFOX	1452 (15.9)	594 (14.7)	769 (17.1)	89 (14.7)
With capecitabine	72 (0.8)	28 (0.7)	>40 (≤5)	≤5
With VEGF inhibitor and capecitabine maintenance	348 (3.8)	152 (3.8)	179 (4.0)	17 (2.8)
With EGFR inhibitor	346 (3.8)	308 (7.6)	19 (0.4)	19 (3.1)
With other	74 (0.8)	52 (1.3)	>15 (≤5)	≤5
FOLFOXIRI	66 (0.7)	25 (0.6)	>30 (≤5)	≤5
With VEGF inhibitor	132 (1.4)	49 (1.2)	65 (1.4)	18 (3.0)
With other	36 (0.4)	19 (0.5)	>10 (≤5)	≤5
Other	440 (4.8)	223 (5.5)	176 (3.9)	41 (6.8)

Abbreviations: CAPEOX, capecitabine and oxaliplatin; EGFR, epithelial growth factor receptor; FOLFIRI, folinic acid, fluorouracil, and irinotecan; FOLFOX, folinic acid, fluorouracil, and oxaliplatin; Mut, mutation; VEGF, vascular endothelial growth factor; WT, wild-type.

^a^ In accordance with the data use agreement, cell sizes less than or equal to 5 were masked. Additional cells in corresponding rows and columns were also approximated to prevent derivation of the masked numbers.

Table 3 shows the results from the multivariable logistic models for receipt of targeted therapies. Compared with patients younger than age 40 years, those aged 80 years or older had significantly lower odds of receiving targeted therapies (EGFR or VEGF inhibitors in patients with *RAS*-WT tumors: adjusted odds ratio [aOR], 0.53; 95% CI, 0.36-0.79 and VEGF inhibitors in patients with RAS-Mut tumors: aOR, 0.62; 95% CI, 0.42-0.90). Similarly, compared with patients with an ECOG score of 0 or 1, lower odds of receiving EGFR inhibitors or VEGF inhibitors were noted in patients with an ECOG score of 2 (aOR, 0.74; 95% CI, 0.57-0.97), 3 or 4 (aOR, 0.39; 95% CI, 0.24-0.63), and missing (aOR, 0.79; 95% CI, 0.68-0.91). As well, compared with patients with colon cancer, those with rectal cancer had significantly lower odds of receiving EGFR or VEGF inhibitor therapy (aOR, 0.59; 95% CI, 0.50-0.68). Conversely, compared with White patients, Black patients had significantly higher odds of receiving targeted therapies (aOR, 1.41; 95% CI, 1.10-1.83). Among patients with *RAS*-Mut tumors, we observed similar associations between receipt of VEGF inhibitor therapy and older age, ECOG scores of 2 or missing, and tumor site (rectal vs colon). However, we observed no associations by race and ethnicity or Charlson comorbidity score.

**Table 3.** Factors Associated With Receipt of Targeted Therapy, Stratified by *RAS*-Mut Status^a^

Characteristic	Adjusted odds ratio (95% CI)	
	Among patients with *RAS*-WT tumors, odds of receiving EGFR or VEGF inhibitors^b^	Among patients with *RAS*-Mut tumors, odds of receiving VEGF inhibitors^c^
Age, y		
<40	1 [Reference]	1 [Reference]
40-49	1.36 (0.98-1.89)	1.03 (0.74-1.44)
50-59	1.27 (0.94-1.72)	1.30 (0.95-1.77)
60-69	1.27 (0.94-1.72)	1.19 (0.87-1.61)
70-79	1.10 (0.80-1.50)	1.03 (0.75-1.42)
≥80	0.53 (0.36-0.79)	0.62 (0.42-0.90)
Race and ethnicity		
African American/Black	1.41 (1.10-1.83)	1.19 (0.98-1.43)
Asian	1.13 (0.78-1.67)	1.31 (0.89-1.95)
Hispanic/Latinx	1.03 (0.80-1.34)	1.02 (0.82-1.29)
White	1 [Reference]	1 [Reference]
Other	0.89 (0.70-1.13)	1.07 (0.85-1.36)
Missing	0.83 (0.66-1.05)	0.98 (0.78-1.22)
Sex		
Female	0.93 (0.82-1.07)	0.94 (0.83-1.06)
Male	1 [Reference]	1 [Reference]
ECOG score		
0 or 1	1 [Reference]	1 [Reference]
2	0.74 (0.57-0.97)	0.66 (0.52-0.84)
3 or 4	0.39 (0.24-0.63)	0.70 (0.38-1.30)
Missing	0.79 (0.68-0.91)	0.66 (0.58-0.76)
Charlson Comorbidity Index score		
≤Mean +1 SD	1 [Reference]	1 [Reference]
>Mean +1 SD	0.98 (0.80-1.19)	1.11 (0.92-1.35)
Stage		
IV	1 [Reference]	1 [Reference]
IVA	1.11 (0.94-1.31)	0.97 (0.83-1.13)
IVB	1.10 (0.90-1.33)	1.16 (0.98-1.37)
IVC	1.13 (0.73-1.77)	0.72 (0.50-1.04)
Tumor site		
Colon	1 [Reference]	1 [Reference]
Rectum	0.59 (0.50-0.68)	0.70 (0.60-0.81)
MMR status		
MSS	1 [Reference]	1 [Reference]
MSI	0.70 (0.47-1.03)	0.78 (0.51-1.21)
Unknown/missing	0.91 (0.78-1.06)	0.89 (0.77-1.03)

Abbreviations: ECOG, Eastern Cooperative Oncology Group; EGFR, epithelial growth factor receptor; MMR, mismatch repair deficiency; MSI, microsatellite instable; MSS, microsatellite stable; Mut, mutation; VEGF, vascular endothelial growth factor; WT, wild-type.

^a^ Multivariable models coadjusted for the factors in the table.

^b^ Receipt of either EGFR inhibitors or VEGF inhibitors, compared with neither.

^c^ Receipt of VEGF inhibitors, compared with those who received EGFR inhibitors or no targeted therapy.

Table 4 presents the results from the multivariable Cox proportional hazards models, and the Figure and eFigure 2 in Supplement 1 present the adjusted survival curves associated with receipt of targeted therapies with (eFigure 2 in Supplement 1) or without (Figure) chemotherapy backbone.

**Table 4.** Association Between Receipt of Therapy and Survival, Stratified by *RAS*-Mut Status

Characteristic	aHR (95% CI)^a^		
	Patients with *RAS*-WT tumors regardless of *BRAF*-Mut status (n = 4034)	Patients with *RAS*-Mut tumors regardless of *BRAF*-Mut status (n = 4495)	Patients with *BRAF*-Mut tumors (n = 605)
Deaths, No.	2401	2877	407
Neither	1 [Reference]	1 [Reference]	1 [Reference]
EGFR inhibitor	0.85 (0.74-0.98)	0.83 (0.58-1.18)	0.74 (0.47-1.18)
VEGF inhibitor	1.00 (0.91-1.11)	1.01 (0.93-1.10)	0.79 (0.62-1.00)
Chemotherapy backbone regimens			
FOLFOX + VEGF inhibitor	1 [Reference]	1 [Reference]	1 [Reference]
Capecitabine	1.02 (0.86-1.20)	1.08 (0.94-1.25)	1.16 (0.74-1.81)
CAPEOX	0.95 (0.71-1.27)	0.79 (0.60-1.05)	0.78 (0.31-1.99)
With VEGF inhibitor	0.67 (0.53-0.85)	0.95 (0.78-1.16)	1.06 (0.61-1.85)
FOLFIRI	1.59 (1.15-2.18)	1.94 (1.57-2.40)	1.50 (0.82-2.75)
With VEGF inhibitor	1.04 (0.84-1.27)	1.31 (1.12-1.53)	1.11 (0.70-1.76)
FOLFOX	0.85 (0.75-0.97)	0.94 (0.84-1.05)	1.42 (1.05-1.92)
With VEGF inhibitor + capecitabine maintenance	0.61 (0.49-0.75)	0.68 (0.56-0.83)	0.52 (0.28-0.98)
With EGFR inhibitor	0.79 (0.66-0.94)	0.77 (0.42-1.40)	0.86 (0.46-1.62)

Abbreviations: aHR, adjusted hazard ratio; CAPEOX, capecitabine and oxaliplatin; EGFR, epithelial growth factor receptor; FOLFIRI, folinic acid, fluorouracil, and irinotecan; FOLFOX, folinic acid, fluorouracil, and oxaliplatin; Mut, mutation; VEGF, vascular endothelial growth factor; WT, wild-type.

^a^ Models adjusted for other first-line therapy, mismatch repair deficiency, age, race and ethnicity, sex, tumor site, Charlson comorbidity score, payer type, Eastern Cooperative Oncology Group score, stage.

**Figure.**
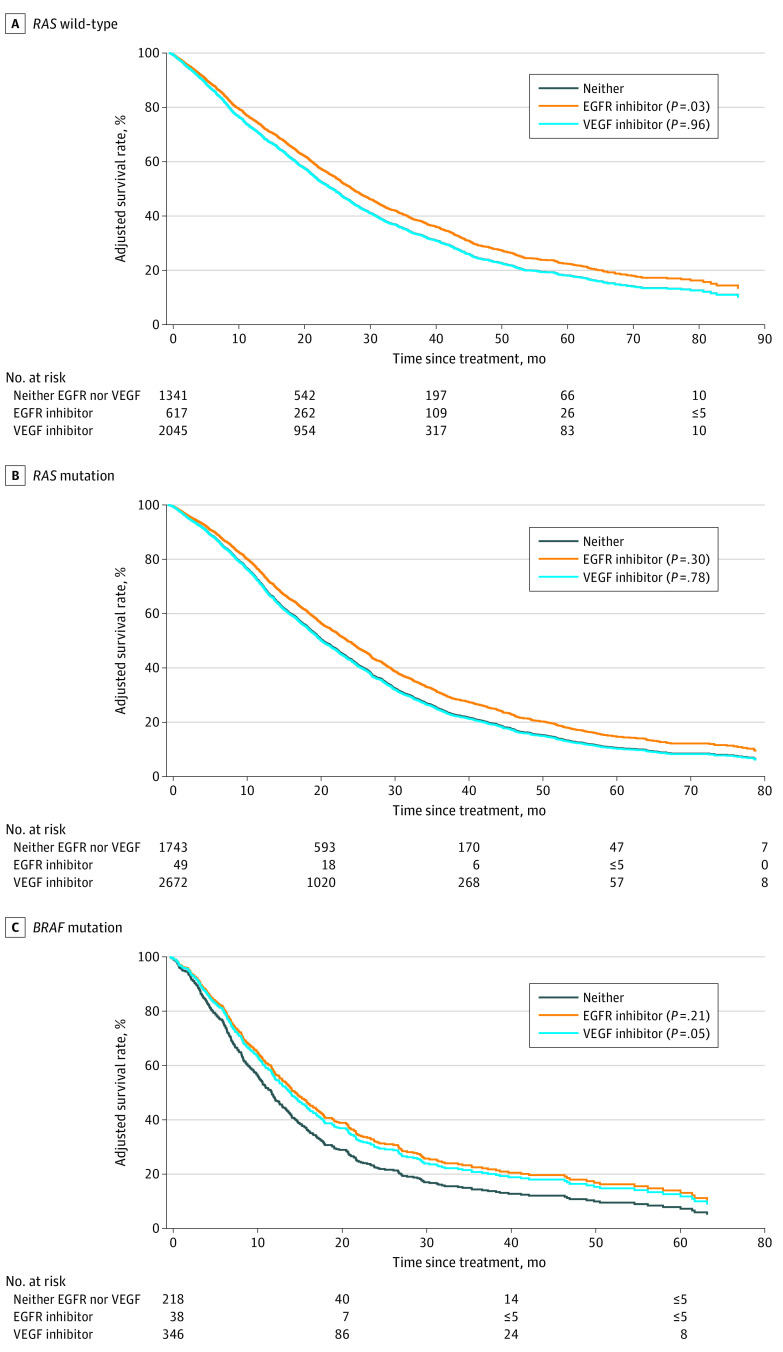
Adjusted Survival Between Targeted Therapy, Stratified by Mutation Status Neither treatment served as the reference category. In accordance with the data use agreement, cells including 5 patients or fewer were masked. In addition, patients who died in the same month in which they received first-line therapy were not included in the survival data (n = 65).

Regardless of chemotherapy backbone, and adjusting for patient and tumor characteristics, the only instance in which we observed a clear survival advantage was with receipt of EGFR inhibitors (compared with neither EGFR inhibitors nor VEGF inhibitors) in patients with *RAS*-WT tumors (adjusted hazard ratio [aHR], 0.85; 95% CI, 0.74-0.98). However, we found no survival benefit from the use of VEGF inhibitors among patients with *RAS*-WT (aHR, 1.00; 95% CI, 0.91-1.11) or *RAS*-Mut (aHR; 1.01; 95% CI, 0.93-1.10) tumors (Figure, Table 4). In addition, in patients with *RAS-*WT tumors, certain targeted therapies and chemotherapy backbone combinations, including CAPEOX (capecitabine and oxaliplatin) + VEGF inhibitors (aHR, 0.67; 95% CI, 0.53-0.85), FOLFOX (aHR, 0.85; 95% CI, 0.75-0.97), FOLFOX + VEGF inhibitors + maintenance capecitabine (aHR, 0.61; 95% CI, 0.49-0.75), and FOLFOX + EGFR inhibitors (aHR, 0.79; 95% CI, 0.66-0.94), were associated with improved survival compared with those who received FOLFOX + VEGF inhibitors. Conversely, FOLFIRI therapy was associated with an increased hazard of death, most notably among those with *RAS*-Mut tumors (aHR, 1.94; 95% CI, 1.57-2.40) and *RAS*-WT tumors (aHR, 1.59; 95% CI, 1.15-2.18) eFigure 2 in Supplement 1; Table 4).

### Sensitivity Analyses

We conducted the following sensitivity analyses. First, to account for potential selection bias, we conducted our survival analyses using inverse probability weighting (eTable 2 in Supplement 1),^26^ which showed a slight increase in the aHR associated with receipt of EGFR inhibitors that was not statistically significant (aHR, 0.86; 95% CI, 0.74-1.01). Second, we examined patterns associated with receipt of VEGF inhibitors in patients with *RAS*-Mut tumors with their mutation status identified before first-line therapy (n = 1875). Although the results were similar to those in patients who received first-line treatment after having their mutation status identified, some of the associations with older age and tumor site were no longer statistically significant (eTable 3 in Supplement 1). Third, we examined the odds of receiving VEGF inhibitor therapy among patients with *RAS*-Mut tumors, after excluding those who received EGFR inhibitors, and the results were similar (eTable 4 in Supplement 1). Fourth, we conducted survival analysis in patients with *RAS*-WT tumors, after excluding those with *NRAS* and *BRAF* unknown status, and the results showed that, although the point estimate for the aHR associated with EGFR inhibitors was similar to the previous analysis (0.85), the upper limit of the 95% CI increased to 1.05 (eTable 5 in Supplement 1).

## Discussion

To our knowledge, this was one of the largest observational studies to date, and our findings showed that receipt of EGFR inhibitor therapy, regardless of chemotherapy backbone, was associated with improved survival among patients with mCRC with *RAS*-WT tumors. Our favorable results relative to EGFR inhibitors are in line with data from RCTs.^10^ However, we did not observe survival benefits with receipt of VEGF inhibitors in patients with *RAS*-WT or *RAS*-Mut tumors. These results are inconsistent with RCT data^27^; however, at least 1 observational study from an earlier period^28^ reported the lack of survival benefits associated with VEGF inhibitor therapy.

We highlight the following noteworthy findings. First, 36.6% of the patients did not receive any targeted therapies. Factors associated with lower odds of receiving such treatment included older age (≥80 years) and higher ECOG scores; given the increased risk with VEGF inhibitor therapy to develop certain complications (eg, bowel perforation, arterial thrombosis, and bleeding),^29,30^ there may be reluctance to treat older and vulnerable patients with targeted therapies. As well, this reflects a more judicious choice of therapy in patients who may draw limited benefits, given how costly these treatments are and the uncertainties surrounding the cost-effectiveness of these therapies.^31,32^ Second, while we anticipated that, compared with White patients, historically marginalized patient populations would have lower odds of receiving targeted therapies, especially in light of extensive prior documentation of higher mortality among Black patients with mCRC,^33,34,35,36,37^ our findings unexpectedly showed that Black patients had higher odds of receiving such treatment. We note, however, that because of absent data on tumor sidedness, we were unable to determine the appropriateness of the treatment received with respect to EGFR inhibitors. Third, our findings showed that targeted therapies with certain combinations of chemotherapy backbone were associated with improved overall survival compared with FOLFOX + VEGF inhibitors, the most commonly observed treatment administered in our study across the mutations.

### Strengths and Limitations

Our study has multiple strengths. First, in addition to being what is, to our knowledge, one of the largest observational studies to date, the data were obtained from more than 800 oncology clinics across the US,^22^ and mostly from community practices, resulting in a demographically and clinically more diverse study population than that in RCTs.^29,38,39,40^ Second, our study period spanned more than 7 years, including the first 3 months of 2020, making it one of the most recent studies on targeted therapies to date. In addition, our data source consisted of electronic health records, which, despite methodologic challenges,^41,42^ offer several advantages, including longitudinal clinical data for patients of all ages and all payer types.^42^

This study has limitations. First, we lacked critical data on several key measures, including tumor sidedness, we had sparse data on comorbidity burden, and we noted high rates of missingness in such critical measures as ECOG score and insurance status, making it difficult for us to adequately adjust for selection bias. Nonetheless, applying inverse probability weighting to our data did not change our results. Second, with over 94% of our study population being treated in community practice settings, we were unable to compare our outcomes across academic and community practice settings. Third, the focus in our study was on first-line therapy and did not account for second-line therapy, switches in treatment, or interrupted cycles—all of which may have affected our assessment of targeted therapies’ associations with improving overall survival. Fourth, we were unable to determine specific mutations (eg, *BRAF* V600 mutation), given that we only had dichotomous reporting of *RAS* and *BRAF*, which have previously been shown to be associated with response to therapy and outcomes.^43,44^ Lastly, we did not conduct analyses by receipt of *BRAF* inhibitors, given the small number of patients in that treatment category.

## Conclusions

In this cohort study, with the exception of EGFR inhibitor therapy, which was associated with improved survival in patients with *RAS-*WT tumors, our findings showed mixed results on survival benefits associated with targeted therapies. With this being an observational study, the data reflect routine clinical care received by a diverse patient population, including racial and ethnic minoritized groups and those with compromised health status, attesting to the generalizability of our findings. In addition, given that some of our results differ from those of RCTs, our study highlights the importance of analyzing outcomes using data originating from routine clinical practice.

## Notes

Supplement 1. **eFigure 1.** CONSORT Diagram of the Study Population **eFigure 2.** Adjusted Survival Between Targeted Therapy With Chemotherapy Backbone, Stratified by Mutation Status **eTable 1.** Mutation Status of Other Mutations Within the Three Mutually Exclusive Categories **eTable 2.** Association Between Receipt of Therapy and Survival, Stratified by *RAS* Mutation Status, After Applying Inverse Probability Weighting **eTable 3.** Factors Associated With Receipt of VEGFi Patients With *RAS*-Mut Tumors, With Their Mutation Status Identified Before First-Line Therapy **eTable 4.** Factors Associated With Receipt of VEGFi Among *RAS*-Mut Patients, After Excluding Patients Who Received EGFRi (n=4,445) **eTable 5.** Association Between Receipt of Therapy and Survival, Among *RAS*-WT Patients With Known *BRAF* and *NRAS* Negative Status (n=2,161)

Supplement 2.
Data Sharing Statement
