# Supplementary material for: Receipt of Targeted Therapy and Survival Outcomes in Patients With Metastatic Colorectal Cancer
Source: JAMA Netw Open. 2023 Jan 19;6(1):e2250030. doi: 10.1001/jamanetworkopen.2022.50030 (PMC9857024; doi:10.1001/jamanetworkopen.2022.50030)
Supplement: Supplement 1. — eFigure 1. CONSORT Diagram of the Study Population eFigure 2. Adjusted Survival Between Targeted Therapy With Chemotherapy Backbone, Stratified by Mutation Status eTable 1. Mutation Status of Other Mutations Within the Three Mutually Exclusive Categories eTable 2. Association Between Receipt of Therapy and Survival, Stratified by RAS Mutation Status, After Applying Inverse Probability Weighting eTable 3. Factors Associated With Receipt of VEGFi Patients With RAS-Mut Tumors, With Their Mutation Status Identified Before First-Line Therapy eTable 4. Factors Associated With Receipt of VEGFi Among RAS-Mut Patients, After Excluding Patients Who Received EGFRi (n=4,445) eTable 5. Association Between Receipt of Therapy and Survival, Among RAS-WT Patients With Known BRAF and NRAS Negative Status (n=2,161) [file jamanetwopen-e2250030-s001.pdf]

# Supplemental Online Content

Koroukian SM, Booker BD, Vu L, et al. Receipt of targeted therapy and survival outcomes in patients with metastatic colorectal cancer. *JAMA Netw Open.* 2023;6(1):e2250030. doi:10.1001/jamanetworkopen.2022.50030

**eFigure 1.** CONSORT Diagram of the Study Population

**eFigure 2.** Adjusted Survival Between Targeted Therapy With Chemotherapy Backbone, Stratified by Mutation Status

**eTable 1.** Mutation Status of Other Mutations Within the Three Mutually Exclusive Categories

**eTable 2.** Association Between Receipt of Therapy and Survival, Stratified by *RAS* Mutation Status, After Applying Inverse Probability Weighting

**eTable 3.** Factors Associated With Receipt of VEGFi Patients With *RAS*-Mut Tumors, With Their Mutation Status Identified Before First-Line Therapy

**eTable 4.** Factors Associated With Receipt of VEGFi Among *RAS*-Mut Patients, After Excluding Patients Who Received EGFRi (n=4,445)

**eTable 5.** Association Between Receipt of Therapy and Survival, Among *RAS*-WT Patients With Known *BRAF* and *NRAS* Negative Status (n=2,161)

This supplemental material has been provided by the authors to give readers additional information about their work.

© 2023 Koroukian SM et al. *JAMA Network Open*.

**eFigure 1: Consort Diagram of the Study Population**

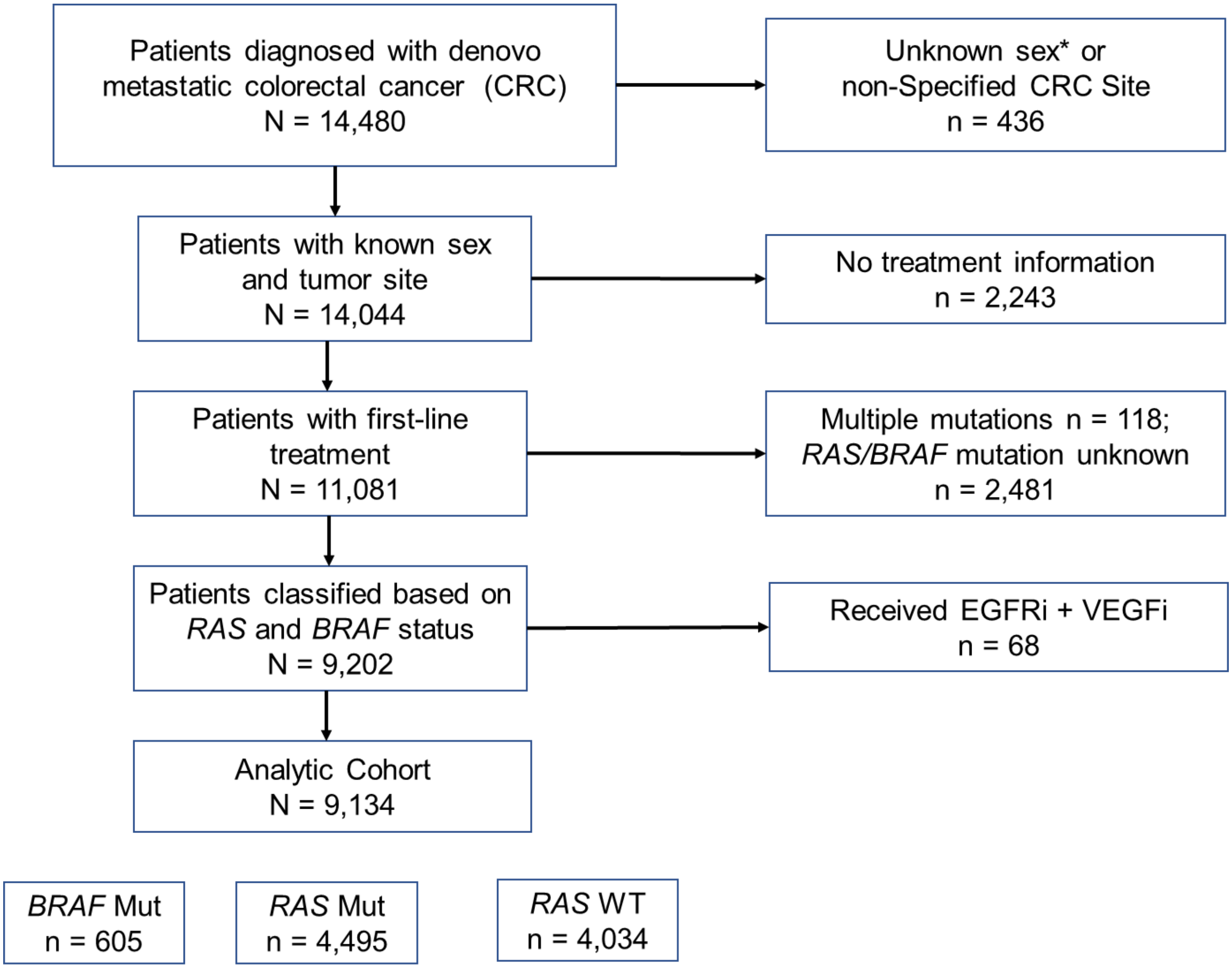


* Unknown sex: n <= 5

© 2023 Koroukian SM et al. *JAMA Network Open*.

eFigure 2: Adjusted survival between targeted therapy with chemotherapy backbone, stratified by mutation status

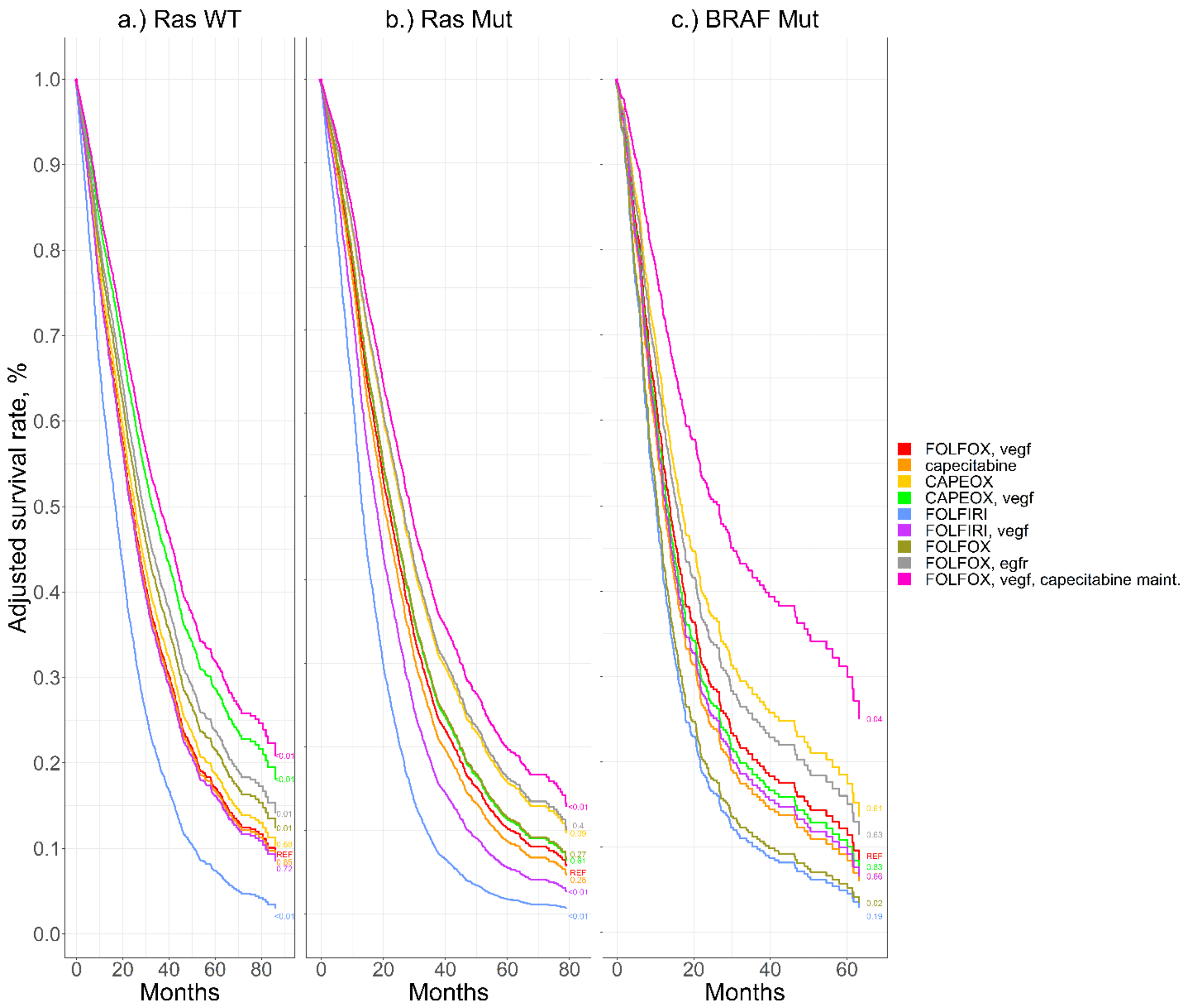


| | Patients with RAS-WT tumors | | Patients with RAS-Mut tumors | | Patients with BRAF-Mut tumors | |
|---|---|---|---|---|---|---|
| | **n (death)** | **Median Survival (95% CI)** | **n (death)** | **Median Survival (95% CI)** | **n (death)** | **Median Survival (95% CI)** |
| FOLFOX, vegf | 1366<br>828 | 24.0 (22.6, 25.6) | 1746<br>1118 | 21.2 (20.1, 22.2) | 228<br>157 | 13.7 (12.0, 15.5) |
| FOLFOX | 594<br>324 | 28.5 (25.5, 30.8) | 769<br>461 | 21.8 (19.5, 24.1) | 89<br>63 | 10.6 (7.3, 15.8) |
| FOLFOX, egfr | 308<br>156 | 33.2 (29.0, 40.1) | 19<br>11 | 32.2 (17.8, NA) | 19<br>11 | 14.7 (10.0, NA) |
| capecitabine | 273<br>183 | 22.5 (20.2, 27.5) | 351<br>240 | 19.9 (18.3, 23.4) | 37<br>25 | 9.6 (8.4, 23.8) |
| FOLFIRI | 67<br>41 | 15.9 (12.0, 31.0) | 117<br>94 | 12.9 (10.5, 16.4) | 18<br>12 | 13.5 (4.8, NA) |
| FOLFIRI, vegf | 161<br>105 | 25.8 (20.3, 30.3) | 265<br>190 | 18.2 (15.5, 19.9) | 30<br>21 | 13.4 (9.3, 29.2) |

© 2023 Koroukian SM et al. *JAMA Network Open*.

| | | | | | | |
|---|---|---|---|---|---|---|
| CAPEOX, vegf | 159<br>72 | 31.0 (28.0, 44.3) | 179<br>112 | 20.8 (17.9, 25.7) | 22<br>15 | 13.3 (11.0, NA) |
| FOLFOX, vegf, capecitabine maint. | 152<br>91 | 33.5 (29.7, 42.9) | 179<br>113 | 30.2 (26.4, 33.6) | 17<br>11 | 24.4 (20.6, NA) |
| CAPEOX | 89<br>48 | 26.0 (21.5, 38.3) | 96<br>53 | 24.9 (18.7, 36.6) | 11<br><= 5 | NA (4.0, NA) |

In accordance with our data use agreement, cell sizes less than or equal to 5 were masked.

© 2023 Koroukian SM et al. *JAMA Network Open*.

eTable 1: Mutation status of other mutations within the three mutually exclusive categories

| | |
|---|---|
| Ras Mut<br>n = 4,495 | BRAF Negative n = 2,935<br>BRAF Unknown n = 1,560 |
| BRAF Mut<br>n = 605 | Ras Mut Negative n = 565<br>Ras Mut Unknown n = 40 |
| Ras WT<br>n = 4,034 | KRAS NRAS and BRAF neg n = 2,161<br>NRAS & BRAF unknown n = 1,873 |

© 2023 Koroukian SM et al. *JAMA Network Open*.

eTable 2: Association between receipt of therapy and survival, stratified by *RAS* mutation status, after applying inverse probability weighting

| | Patients with *RAS*-WT tumors<br><br>aHR (95% CI)*<br>N=4,034<br>Deaths=2,401 | Patients with *RAS*-Mut tumors<br><br>aHR (95% CI)*<br>N=4,495<br>Deaths=2,877 | Patients with *BRAF*-Mut tumors<br><br>aHR (95% CI)*<br>N=605<br>Deaths=407 |
|---|---|---|---|
| Neither | 1.0 | 1.0 | 1.0 |
| EGFRi | 0.86 (0.74-1.01) | 0.92 (0.53-1.58) | 0.84 (0.54-1.30) |
| VEGFi | 1.03 (0.93-1.14) | 1.02 (0.94-1.12) | 0.84 (0.67-1.04) |
| **Chemotherapy Backbone Regimes** | | | |
| FOLFOX+VEGFi | 1.0 | 1.0 | 1.0 |
| Capecitabine | 0.99 (0.79-1.24) | 1.05 (0.88-1.26) | 0.91 (0.49-1.68) |
| CAPEOX | 0.77 (0.5-1.2) | 0.78 (0.54-1.12) | 0.34 (0.06-1.85) |
| CAPEOX+VEGFi | 0.63 (0.48-0.83) | 1.05 (0.85-1.29) | 1.20 (0.65-2.22) |
| FOLFIRI | 1.51 (1.01-2.29) | 2.02 (1.50-2.71) | 1.38 (0.64-2.99) |
| FOLFIRI+VEGFi | 1.07 (0.85-1.36) | 1.29 (1.11-1.49) | 1.00 (0.58-1.71) |
| FOLFOX | 0.79 (0.68-0.91) | 0.92 (0.81-1.03) | 1.42 (1.05-1.91) |
| FOLFOX+VEGFi+capecitabine maint. | 0.57 (0.46-0.72) | 0.71 (0.57-0.88) | 0.15 (0.04-0.56) |
| FOLFOX+EGFRi | 0.79 (0.65-0.97) | 0.78 (0.43-1.43) | 0.93 (0.41-2.13) |

*aHR and (95% CI): Adjusted Hazard Ratio and (95% Confidence Interval)

© 2023 Koroukian SM et al. *JAMA Network Open*.

eTable 3: Factors associated with receipt of VEGFi patients with *RAS*-Mut tumors, with their mutation status identified before first line therapy

| | **No** | **Yes** | **OR (multivariable)** |
|---|---|---|---|
| Age categories: | | | |
| <40 | 36 (45.6) | 43 (54.4) | 1.0 |
| 40-49 | 96 (42.1) | 132 (57.9) | 1.17 (0.69-1.98) |
| 50-59 | 175 (37.7) | 289 (62.3) | 1.40 (0.85-2.29) |
| 60-69 | 200 (36.5) | 348 (63.5) | 1.53 (0.93-2.51) |
| 70-79 | 171 (39.1) | 266 (60.9) | 1.41 (0.85-2.34) |
| 80+ | 66 (55.5) | 53 (44.5) | 0.71 (0.39-1.28) |
| Race: | | | |
| White | 473 (42.3) | 644 (57.7) | 1.0 |
| Black | 88 (32.2) | 185 (67.8) | 1.52 (1.14-2.03) |
| Asian | 18 (32.1) | 38 (67.9) | 1.57 (0.88-2.88) |
| Hispanic | 46 (30.9) | 103 (69.1) | 1.61 (1.11-2.36) |
| Other Race | 55 (43.3) | 72 (56.7) | 0.99 (0.68-1.44) |
| Missing | 64 (41.8) | 89 (58.2) | 1.03 (0.72-1.47) |
| Sex: | | | |
| Female | 342 (39.0) | 534 (61.0) | 1.08 (0.89-1.31) |
| Male | 402 (40.2) | 597 (59.8) | 1.0 |
| ECOG Score: | | | |
| 1 | 307 (32.1) | 648 (67.9) | 1.0 |
| 2 | >60 (>48.0) | >60 (>51.0) | 0.51 (0.35-0.75) |
| 3 | >10 (68.8) | <= 5 (31.2) | 0.21 (0.06-0.59) |
| Unknown | 364 (47.0) | 411 (53.0) | 0.53 (0.44-0.65) |
| Charlson Score: | | | |
| <= mean + 1Sd | 660 (39.7) | 1004 (60.3) | 1.0 |
| > mean + 1Sd | 84 (39.8) | 127 (60.2) | 1.06 (0.78-1.45) |
| Stage: | | | |
| IV | 186 (39.5) | 285 (60.5) | 1.0 |
| IVA | 323 (40.6) | 472 (59.4) | 0.89 (0.70-1.13) |
| IVB | 203 (37.5) | 338 (62.5) | 1.09 (0.84-1.42) |
| IVC | 32 (47.1) | 36 (52.9) | 0.62 (0.36-1.06) |
| Tumor Site: | | | |
| Colon | 628 (39.5) | 963 (60.5) | 1.0 |
| Rectum | 116 (40.8) | 168 (59.2) | 0.93 (0.71-1.21) |
| MMR Status: | | | |
| MSS | 522 (38.6) | 832 (61.4) | 1.0 |
| MSI | 22 (48.9) | 23 (51.1) | 0.63 (0.34-1.16) |
| Unknown/Missing | 200 (42.0) | 276 (58.0) | 0.89 (0.71-1.12) |

© 2023 Koroukian SM et al. *JAMA Network Open*.

In accordance with our data use agreement, cell sizes less than or equal to 5 were masked. Additional cells in corresponding rows and columns were approximated to prevent derivation of the masked cells.

© 2023 Koroukian SM et al. *JAMA Network Open*.

eTable 4: Factors associated with receipt of VEGFi among *RAS*-Mut patients, after excluding patients who received EGFRi (n=4,445)

| | Adjusted Odds Ratios 95% Confidence Interval |
|---|---|
| Age categories: | |
| <40 | 1.0 |
| 40-49 | 1.01 (0.72-1.40) |
| 50-59 | 1.28 (0.93-1.75) |
| 60-69 | 1.16 (0.85-1.59) |
| 70-79 | 1.01 (0.73-1.39) |
| 80+ | 0.63 (0.43-0.91) |
| Race: | |
| White | 1.0 |
| Black | 1.19 (0.98-1.44) |
| Asian | 1.36 (0.92-2.05) |
| Hispanic | 1.01 (0.80-1.27) |
| Other Race | 1.06 (0.84-1.35) |
| Missing | 1.00 (0.79-1.25) |
| Sex: | |
| Female | 0.94 (0.83-1.06) |
| Male | 1.0 |
| ECOG Score: | |
| 1 | 1.0 |
| 2 | 0.64 (0.50-0.82) |
| 3 | 0.71 (0.38-1.34) |
| Missing | 0.67 (0.59-0.77) |
| Comorbidity: Charlson Score | |
| <= mean + 1Sd | 1.0 |
| > mean + 1Sd | 1.13 (0.93-1.37) |
| Stage: | |
| IV | 1.0 |
| IVA | 0.97 (0.83-1.14) |
| IVB | 1.19 (1.00-1.41) |
| IVC | 0.78 (0.54-1.14) |
| Tumor Site: | |
| Colon | 1.0 |
| Rectum | 0.70 (0.60-0.81) |
| MMR status: | |
| MSS | 1.0 |
| MSI | 0.81 (0.53-1.26) |
| Unknown | 0.87 (0.76-1.01) |

© 2023 Koroukian SM et al. *JAMA Network Open*.

eTable 5: Association between receipt of therapy and survival, among *RAS*-WT patients with known BRAF and NRAS negative status (n=2,161)

| | Patients with *RAS*-WT & known *BRAF* and *NRAS* negative status<br>AHR (95% CI)*<br>N=2,161<br>Deaths=1,072 |
|---|---|
| Neither | 1.0 |
| EGFRi | 0.85 (0.69-1.05) |
| VEGFi | 1.05 (0.91-1.23) |
| **Chemotherapy Backbone Regimes** | |
| FOLFOX+VEGFi | 1.0 |
| Capecitabine | 1.11 (0.86-1.44) |
| CAPEOX | 0.80 (0.52-1.23) |
| CAPEOX+VEGFi | 0.47 (0.33-0.69) |
| FOLFIRI | 1.19 (0.75-1.89) |
| FOLFIRI+VEGFi | 1.03 (0.75-1.41) |
| FOLFOX | 0.80 (0.66-0.97) |
| FOLFOX+VEGFi+capecitabine maint. | 0.67 (0.49-0.93) |
| FOLFOX+EGFRi | 0.74 (0.58-0.95) |

© 2023 Koroukian SM et al. *JAMA Network Open*.
