# Supplementary material for: Receipt of Targeted Therapy and Survival Outcomes in Patients With Metastatic Colorectal Cancer
Source: JAMA Netw Open. 2023 Jan 19;6(1):e2250030. doi: 10.1001/jamanetworkopen.2022.50030 (PMC9857024; doi:10.1001/jamanetworkopen.2022.50030)
Supplement: Supplement 2. — Data Sharing Statement [file jamanetwopen-e2250030-s002.pdf]

Data Sharing Statement

Koroukian. Receipt of Targeted Therapy and Survival Outcomes in Patients With Metastatic Colorectal Cancer. *JAMA Netw Open*. Published January 19, 2023.
doi:10.1001/jamanetworkopen.2022.50030

Data

Data available: The data for this study were provided to the authors by Flatiron Health, Inc. and are not publicly available. These deidentified data may be made available upon request, and are subject to a license agreement with Flatiron Health; interested researchers should contact DataAccess@flatiron.com to determine licensing terms.
